# Rapid *in vivo* evaluation system for cholestasis-related genes in mice with humanized bile acid profiles

**DOI:** 10.1097/HC9.0000000000000382

**Published:** 2024-03-22

**Authors:** Kihiro Wakasa, Ryutaro Tamura, Shuhei Osaka, Hajime Takei, Akihiro Asai, Hiroshi Nittono, Hiroyuki Kusuhara, Hisamitsu Hayashi

**Affiliations:** 1 Laboratory of Molecular Pharmacokinetics, Graduate School of Pharmaceutical Science, The University of Tokyo, Tokyo, Japan; 2 Junshin Clinic Bile Acid Institute, Tokyo, Japan; 3 Department of Gastroenterology, and Hepatology, and Nutrition, Cincinnati Children’s Hospital Medical Center, Cincinnati, Ohio, USA; 4 Department of Pediatrics, University of Cincinnati College of Medicine, Cincinnati, Ohio, USA

## Abstract

**Background:**

Pediatric cholestatic liver diseases (Ped-CLD) comprise many ultrarare disorders with a genetic basis. Pharmacologic therapy for severe cases of Ped-CLD has not been established. Species differences in bile acid (BA) metabolism between humans and rodents contribute to the lack of phenocopy of patients with Ped-CLD in rodents and hinder the development of therapeutic strategies. We aimed to establish an efficient *in vivo* system to understand BA-related pathogenesis, such as Ped-CLD.

**Methods:**

We generated mice that express spCas9 specifically in the liver (L-Cas9^Tg/Tg^ [liver-specific Cas9^Tg/Tg^] mice) and designed recombinant adeno-associated virus serotype 8 encoding small-guide RNA (AAV8 sgRNA) targeting *Abcc2*, *Abcb11*, and *Cyp2c70*. In humans, *ABCC2* and *ABCB11* deficiencies cause constitutional hyperbilirubinemia and most severe Ped-CLD, respectively. *Cyp2c70* encodes an enzyme responsible for the rodent-specific BA profile. Six-week-old L-Cas9^Tg/Tg^ mice were injected with this AAV8 sgRNA and subjected to biochemical and histological analysis.

**Results:**

Fourteen days after the injection with AAV8 sgRNA targeting *Abcc2*, L-Cas9^Tg/Tg^ mice exhibited jaundice and phenocopied patients with *ABCC2* deficiency. L-Cas9^Tg/Tg^ mice injected with AAV8 sgRNA targeting *Abcb11* showed hepatomegaly and cholestasis without histological evidence of liver injury. Compared to *Abcb11* alone, simultaneous injection of AAV8 sgRNA for *Abcb11* and *Cyp2c70* humanized the BA profile and caused higher transaminase levels and parenchymal necrosis, resembling phenotypes with *ABCB11* deficiency.

**Conclusions:**

This study provides proof of concept for efficient *in vivo* assessment of cholestasis-related genes in humanized bile acid profiles. Our platform offers a more time- and cost-effective alternative to conventional genetically engineered mice, increasing our understanding of BA-related pathogenesis such as Ped-CLD and expanding the potential for translational research.

## INTRODUCTION

Pediatric cholestatic liver diseases (Ped-CLD) comprise many ultrarare disorders with a genetic basis.^1^ Ped-CLD occurs in one in 2500 newborns in North America, 40% of which are caused by biliary atresia; the rest are classified as genetic cholestatic liver diseases.^2^ Affected patients present with neonatal hepatitis syndrome and are at increased risk for complications of chronic cholestasis, such as sustained intractable itching, jaundice, failure to thrive, and progression to liver failure. In addition, severe pruritus accompanied by sleep disturbance leads to serious mental distress in the patients and their families and significantly reduces their quality of life.^3^ Liver transplantation has been established as the only curative treatment for severe cases of Ped-CLD that symptomatic therapies cannot control. Therefore, developing new, less invasive medical treatments for these diseases is the highest priority.

Bile acid (BA) facilitates intestinal fat absorption through its amphiphilic property and causes systemic actions by farnesoid X receptor signaling.^4^ There are species differences in the hydrophobicity of BA composition between humans and rodents, making it challenging to analyze the pathogenesis of Ped-CLD and develop therapeutic strategies.^5^ *Abcb11* encoding bile salt export pump, which belongs to the ABC transporter family, is localized on the canalicular membrane of hepatocytes and responsible for BAs’ biliary excretion.^6–8^ Biallelic pathogenic variants of *ABCB11* cause progressive familial intrahepatic cholestasis type 2 (PFIC2) in humans, leading to cholestasis and progressive cirrhosis in the first decade of life.^9,10^ *Abcb11* KO mice had milder cholestasis and liver injury than patients with PFIC2. This suggests that different hydrophobicity in BA profiles in humans and rodents may cause the lack of phenocopy of patients with PFIC2 in *Abcb11* KO mice. Recent studies demonstrated that cytochrome P450, family 2, subfamily C, polypeptide 70 (Cyp2c70), a hepatic enzyme unique to rodents, catalyzes the sequential reaction of 6β-hydroxylation and C7-epimerization of chenodeoxycholic acid and generates muricholic acid (MCA), a trihydroxylated, highly hydrophilic type of BA.^11^ MCA is the most abundant in rodents and rare in other animals.^12^ *Cyp2c70* KO in mice makes the BA composition hydrophobic and cytotoxic as in humans and causes hepatic injury.^13,14^ Therefore, *Cyp2c70*-deficient mice may have applications in understanding BA-related pathogenesis, such as Ped-CLD.

**FIGURE 1:**
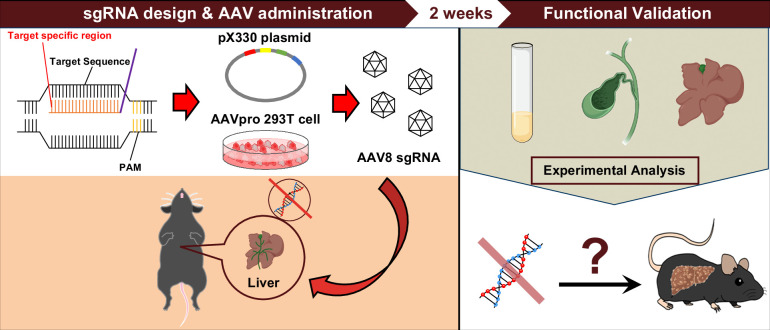
Experimental procedures used in this study.

In this study, we first developed and optimized a rapid genome editing system targeting the liver (Figure 1). Liver-specific Cas9^Tg/Tg^ (L-Cas9^Tg/Tg^ mice) were generated. Recombinant adeno-associated virus serotype 8 encoding small-guide RNA (AAV8 sgRNA) targeting *Abcc2*, a gene responsible for constitutional jaundice, was injected into L-Cas9^Tg/Tg^ mice. After determining the optimal conditions for AAV8 sgRNA administration method and dosage, we tested simultaneous injection of AAV8 sgRNA targeting *Cyp2c70* with AAV8 sgRNA targeting *Abcb11.* The resulting mice were analyzed to evaluate the development of cholestasis and jaundice and the humanization of their BA profile. This platform makes it possible to assess preclinical *in vivo* outcomes within a month: 2 weeks for the production of AAV8 sgRNA targeting a candidate gene and 2 weeks for analysis of L-Cas9^Tg/Tg^ mice injected with the designed AAV8 sgRNA.

## MATERIALS AND METHODS

The reagents used in this study were of analytical grade and are listed in Supplemental Table S1, http://links.lww.com/HC9/A795.

### Animals and diets

All mouse experiments were approved by and performed following the guidelines of the animal experiment committee of the University of Tokyo (permission number: P29-24). Mice were kept in plastic cages in a room maintained at 23.5°C±2.5°C and 52.5%±12.5% relative humidity under a 12-h light:12-h dark cycle. Mice had free access to water and a standard chow diet (D10012G; Research Diets Inc., New Brunswick, NJ).

### Mice generation

B6J.129(B6N)-Gt(ROSA)26Sor^tm1(CAG-cas9*,-EGFP)Fezh^/J mice (Stock No. 026175; The Jackson Laboratory, Bar Harbor, ME)^15^ were crossed with B6.Cg-Speer6-ps1^Tg(Alb-cre)21Mgn^/J mice (Stock No. 003574; The Jackson Laboratory),^16^ and the resulting offspring were mated to generate L-Cas9^Tg/Tg^ mice, which express Cas9 in hepatocytes and cholangiocytes in a Cre recombinase-dependent manner.

### sgRNA design and plasmid construction

SgRNA was designed on Integrated DNA Technologies (https://sg.idtdna.com/pages), and 4 candidates were selected for each target gene based on on-target and off-target scores. The selected sequences were synthesized with the following modifications: forward, 5′-CACCG-(the selected sequence)-3′ and reverse, 5′-AAAC-(the selected sequence)-C-3′ (Eurofins Genomics K. K., Tokyo, Japan). SgRNA sequences for each target gene used in this study are shown in Supplemental Table S2, http://links.lww.com/HC9/A795. Each corresponding sequence pair was annealed to an oligonucleotide as follows: cloned into pX330A or pX330S plasmid (#1000000055; Addgene, Cambridge, MA),^17^ assembled into one segment through golden gate reaction, and subcloned to pAAV-U6-sgRNA-CMV-GFP (#85451; Addgene) through in-fusion reaction.

**Figure:**
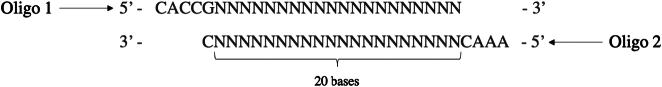


### Cell culture

AAVpro 293T cells (Takara Bio Inc, Shiga, Japan) were maintained in DMEM, high-glucose, pyruvate (Thermo Fisher Scientific, San Jose, CA) supplemented with 10% fetal bovine serum (Thermo Fisher Scientific), and 1% penicillin-streptomycin solution (FUJIFILM Wako Pure Chemical) and cultured at 37°C in 5% CO_2_ at 95% humidity.

### AAV production

AAVpro 293T cells were seeded at 4 × 10^6^ cells per 10-cm culture dish and cultured for 24 h. The culture medium was replaced with DMEM, high-glucose, and pyruvate supplemented with 2% fetal bovine serum and 1% penicillin-streptomycin solution, and transfected with pAAV-U6-sgRNA-CMV-GFP vectors, pAAV2/8 vectors (#112864; Addgene) and pHelper vectors (No. 340202, Cell Biolabs, San Diego, CA). Three days after the transfection, the cells were detached with 500 mM ethylenediaminetetraacetic acid (EDTA, FUJIFILM Wako Pure Chemical) and collected by centrifugation at 1000 × g for 10 min at 4°C. The pellet was treated with an AAV extraction solution (Takara Bio Inc) to collect the AAV8 sgRNA produced. The titer of AAV sgRNA was quantified using the CFX Connect real-time system (Bio-Rad Laboratories, Inc. Hercules, CA) by amplifying Inverted Terminal Repeat 2.^18^

### Animal studies

Male and female L-Cas9^Tg/Tg^ mice aged 6−7 weeks were injected intravenously into the tail vein or intraperitoneally with the prepared AAV8 sgRNA at a range of 0.33 × 10^12^ to 3 × 10^12^ genome copies per mouse. Fourteen days after the AAV8 sgRNA injection, the mice were anesthetized with isoflurane (2%, inhalation anesthesia apparatus), followed by a laparotomy. Blood was collected from the inferior vena cava into heparin-coated tubes. The plasma was separated by centrifugation at 1700 × g for 15 min at 4°C, snap-frozen in liquid nitrogen, and stored at −80°C. Livers were harvested, snap-frozen in liquid nitrogen, and stored at −80°C for biochemical analysis or were processed for quantitative PCR (qPCR) and histological analysis, as described in each section. Bile was collected from the gallbladder, snap-frozen in liquid nitrogen, and stored at −80°C.

### Biochemistry

Plasma alanine aminotransferase (ALT), alkaline phosphatase, and total bilirubin were quantified using DRI-CHEM NX500 (Fuji Film RI Pharma, Tokyo, Japan). BA concentrations and profiles in plasma, liver, and gallbladder bile were evaluated with a Total Bile Acid-Test Wako Kit (FUJIFILM Wako Pure Chemical) and an liquid chromatography with tandem mass spectrometry system.^19^ To analyze the liver, 100 μg liver sections were homogenized in 400 μL 100% ethanol by Tissue Lyser II (QIAGEN, Hilden, Germany) and centrifuged at 1700 × g for 5 min at 4°C. The supernatants were collected, concentrated with centrifugal thickener for 2 h, resuspended in 100 μL 80% ethanol, and subjected to BA analysis. The hydrophobicity index of BAs was calculated as previously.^20^

### Western blotting

Liver tissues were homogenized, and the crude membrane fractions were prepared. The prepared specimens were lysed by Blue Protein Loading Dye (New England Biolabs, Ipswich, MA), loaded onto wells of a 4%–15% Mini-PROTEAN TGX Precast Gel (Bio-Rad Laboratories), and electrophoresed, and transferred onto a polyvinylidene difluoride membrane (Immobilon-P Transfer Membrane; Merck Millipore, Darmstadt, Germany). The membrane was blocked with 5% skim milk (FUJIFILM Wako Pure Chemical) in Tris-buffered saline with 0.1% Tween 20 and incubated with primary antibodies and then the corresponding secondary antibodies. The primary and secondary antibodies used are listed in Supplemental Table S3, http://links.lww.com/HC9/A795. Fusion Solo7S with FusionCapt17 software (Vilber Lourmat, Collégien, France) was used at high resolution and auto exposure to detect immunoreactivity using the WESTAR C ULTRA 2.0 (Cyanagen, Bologna, Italy) and to quantify the intensity of the band indicating each protein.

### Quantitative PCR (qPCR)

According to the manufacturer’s instructions, total RNA was isolated from the mouse liver using ISOGEN II (Nippon gene, Tokyo, Japan). Reverse transcription was performed using ReverTra Ace® qPCR RT Master Mix with gDNA Remover (TOYOBO, Osaka, Japan). The prepared cDNA was analyzed to evaluate mRNA expression levels of target genes. It was determined by real-time qPCR using a CFX Connect real-time system (Bio-Rad Laboratories), CFX Maestro 1.1 software (Bio-Rad Laboratories), and Thunderbird SYBR qPCR Mix (TOYOBO). The primer sequences used in this study are listed in Supplemental Table S4, http://links.lww.com/HC9/A795. The expression of 18S rRNA normalized gene expression for each reaction.

### Histological analysis and immunohistochemistry

A portion of the liver was fixed with 10% Formalin Neutral Buffer Solution (FUJIFILM Wako Pure Chemical) and embedded in paraffin. Three-micrometer paraffin sections were prepared, deparaffinized, rehydrated, stained with Hematoxylin and Eosin, and mounted in Entellan New (Merck, Branchburg, NJ). For immunohistochemistry, the rehydrated paraffin sections were subjected to HistoVT One Solution (pH7.0) (Nacalai Tesque, Kyoto, Japan) in Decloaking Chamber NxGen (Biocare Medical, Concord, CA) for 20 min. Nonspecific antigen binding was blocked with 3% bovine serum albumin in phosphate-buffered saline at room temperature for an hour. Next, the sections were stained with primary antibodies at room temperature for 2 h, followed by Alexa Fluor secondary antibody at room temperature for an hour. The primary and secondary antibodies used are listed in Supplemental Table S3, http://links.lww.com/HC9/A795. After mounting with ProLong Diamond Anti-fade Mountant (Thermo Fisher Scientific), microscopic images were obtained by a Zeiss LSM 880 with Airyscan (Carl Zeiss, Jena, Germany) or Zeiss Axio Scan Z1 (Carl Zeiss) and processed on Zen 3.0 software (Carl Zeiss).

### Statistical analysis

Graphs include means±SEM. In addition, differences between 2 and multiple variables were assessed at the 95% confidence level using Student *t*-tests and ANOVA with post hoc Tukey tests, respectively. The data were analyzed using GraphPad Prism 9.5.1 (GraphPad Software, La Jolla, CA).

## RESULTS

### Optimization of administration conditions of AAV8 sgRNA for hepatic genome editing in L-Cas9^Tg/Tg^ mice

Multidrug resistance-associated protein 2 (Mrp2), encoded by *Abcc2*, belongs to the ABC transporter family, is localized on the canalicular membrane of hepatocytes, and mediates biliary excretion of organic anions including bilirubin glucuronides and glutathione conjugates.^21,22^ In humans, a genetic deficiency in *ABCC2* results in Dubin–Johnson syndrome (DJS), a rare autosomal recessive liver disease. Patients with DJS present with severe jaundice in the neonatal period^23^ and mild to moderate, recurrent jaundice in adolescence and beyond.^1,24^ However, there is no hepatic dysfunction or abnormal liver function tests except for conjugated bilirubin, and the prognosis is excellent. *Abcc2*-knockout mice exhibit mild jaundice, as do patients with DJS.^21^

Initially, to examine the optimal administration of the route of AAV8 sgRNA for hepatic genome editing, male L-Cas9^Tg/Tg^ mice aged 6−7 weeks were injected intravenously and intraperitoneally with AAV8-*Abcc2* sgRNA or its corresponding control (AAV8-nontargeted (NT) sgRNA) at 1 × 10^12^ genome copies. Two weeks after the injection, AAV8-*Abcc2* sgRNA significantly reduced hepatic *Abcc2* expression at mRNA and Mrp2 expression at protein levels to the same degree regardless of the administration route (Supplemental Figure 1A–D, http://links.lww.com/HC9/A794). This was accompanied by mild jaundice and elevated total bilirubin in plasma (Supplemental Figure 1E, F, http://links.lww.com/HC9/A794). Next, to investigate the optimal injection dose of AAV8 sgRNA for hepatic genome editing, male L-Cas9^Tg/Tg^ mice aged 6−7 weeks were injected intraperitoneally with AAV8-*Abcc2* sgRNA at three different doses of 0.3 × 10^12^, 1 × 10^12^, and 3 × 10^12^ genome copies. Hepatic *Abcc2* expression at mRNA and protein levels was evaluated 2 weeks after the injection. AAV8-*Abcc2* sgRNA significantly decreased hepatic *Abcc2* mRNA, but no dose-dependent effect was observed at the doses tested (Supplemental Figure 1G, http://links.lww.com/HC9/A794). This was also the case when hepatic Mrp2 protein was detected by immunohistochemistry (Supplemental Figure 1H, http://links.lww.com/HC9/A794). Western blot analysis showed that at 0.3 × 10^12^ genome copies, hepatic Mrp2 protein was decreased to a lesser extent than the 2 other doses (Supplemental Figure 1I, J, http://links.lww.com/HC9/A794).

Based on these results and the simplicity of the procedure, we decided to intraperitoneally administer AAV sgRNA at 1 × 10^12^ genome copies for subsequent analysis.

### Evaluation of *Abcb11* gene in L-Cas9^Tg/Tg^ mice modified with a human-like BA profile

**FIGURE 2:**
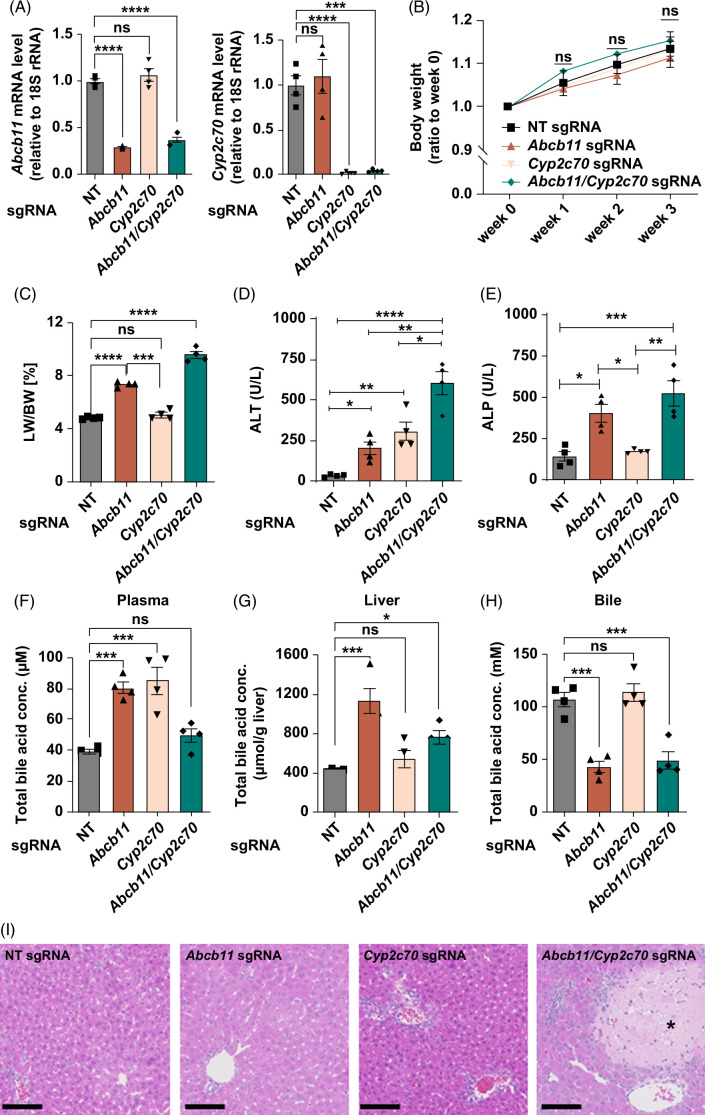
Biochemical and histological analysis of liver function in male L-Cas9^Tg/Tg^ mice injected with AAV8-*Abcb11* sgRNA, AAV8-*Cyp2c70* sgRNA, or both. Male L-Cas9^Tg/Tg^ mice aged 6 weeks were intraperitoneally injected with AAV8-NT sgRNA, AAV8-*Abcb11* sgRNA, AAV8-*Cyp2c70* sgRNA, or both AAV8-*Abcb11* sgRNA and AAV8-*Cyp2c70* sgRNA at 1 × 10^12^ genome copies per mouse (n = 4 in each group). Three weeks after the injection, these mice were sacrificed to collect blood, livers, and gallbladder bile. (A) *Abcb11* and *Cyp2c70* mRNA levels in the liver. The mRNA levels are expressed relative to those of 18S rRNA. (B) Body weight during the experiment. (C–E) Liver weight (C) and plasma levels of ALT (D) and ALP (E) at the laparotomy. (F–H) Total BA levels in plasma (F), liver (G), and gallbladder bile (H). (I) Hematoxylin and Eosin staining of liver section. *lobular necrosis. Scale bar, 100 μm. In (A–H), all data are presented as mean±SEM. *p < 0.05, ***p < 0.001, ****p < 0.0001 by one-way ANOVA with a post hoc Tukey’s test for multiple comparisons. A representative result from 3 independent experiments is shown, each exhibiting a similar pattern of results. Abbreviations: AAV8, adeno-associated virus serotype 8; ALP, alkaline phosphatase; ALT, alanine aminotransferase; BW, body weight; Cyp2c70, cytochrome P450, family 2, subfamily C, polypeptide 70; L-Cas9^Tg/Tg^, liver-specific Cas9^Tg/Tg^; sgRNA, small-guide RNA; LW, liver weight; NT, non-targeted.

To test whether the humanized BA profile better models the patient phenotype of cholestasis and progressive liver injury in *Abcb11-*deficient mice, we performed intraperitoneal single or simultaneous injections of AAV8-*Cyp2c70* sgRNA and AAV8-*Abcb11* sgRNA at 1 × 10^12^ genome copies into male L-Cas9^Tg/Tg^ mice at 6 weeks of age. Three weeks after the injection, hepatic mRNA expression of *Abcb11* and *Cyp2c70* was reduced in the L-Cas9^Tg/Tg^ mice injected with each corresponding AAV8 sgRNA (Figure 2A). There was no significant difference in body weight between each group after AAV8 sgRNA injection (Figure 2B). A plasma marker of hepatocellular injury, ALT, was more elevated in mice co-injected with AAV8-*Cyp2c70* sgRNA and AAV8-*Abcb11* sgRNA than in mice injected with each alone (Figure 2D). Injection of AAV8-*Abcb11* sgRNA alone and co-injection of AAV8-*Cyp2c70* and AAV8-*Abcb11* sgRNA developed hepatomegaly and elevated plasma marker of cholestasis, alkaline phosphatase, while injection of AAV8-*Cyp2c70* sgRNA alone had no such effect (Figure 2C, E). This was supported by measuring total BAs in plasma, liver, and gallbladder bile. The L-Cas9^Tg/Tg^ mice injected with AAV8-*Abcb11* sgRNA alone or both AAV8-*Cyp2c70* sgRNA and AAV8-*Abcb11* sgRNA showed higher BA levels in the liver and lower BA levels in gallbladder bile than those injected with AAV8-NT sgRNA alone, indicating cholestatic conditions (Figure 2F–H). This was not the case for the L-Cas9^Tg/Tg^ mice injected with AAV8-*Cyp2c70* sgRNA. The lower plasma levels of BA in the L-Cas9^Tg/Tg^ mice injected with both AAV8-*Cyp2c70* sgRNA and AAV8-*Abcb11* sgRNA than those with AAV8-*Abcb11* sgRNA alone could be explained by the fact that the plasma levels of C4, an intermediate metabolite for BA synthesis from cholesterol in hepatocytes, was detectable only in the L-Cas9^Tg/Tg^ mice injected with both AAV8-*Cyp2c70* sgRNA and AAV8-*Abcb11* sgRNA (Supplemental Figure 2, http://links.lww.com/HC9/A794). This suggests that *Abcb11* and *Cyp2c70* double knockdown causes accumulation of hydrophobic BA that have high affinity to the bile acid nuclear receptor farnesoid X receptor and negatively regulate bile acid synthesis, suppressing hepatic BA synthesis and reducing plasma bile acid concentration.

Liver histological analysis showed diffuse patchy necrotic lesions and sporadic injury in intralobular bile ducts in mice injected with both AAV8-*Cyp2c70* sgRNA and AAV8-*Abcb11* sgRNA, reflecting the results of plasma ALT concentration (Figure 2I). By contrast, no apparent features of liver injury were observed in the other groups. The necrotic lesions are similar to the injury pattern reported in previous studies in which *Abcb11* KO mice were overloaded with cholic acids.^25,26^ Together, these results indicate that the simultaneous knockdown of hepatic *Abcb11* and *Cyp2c70* caused cholestasis and hepatocellular injury in mice without overloading hydrophobic BAs.

**FIGURE 3:**
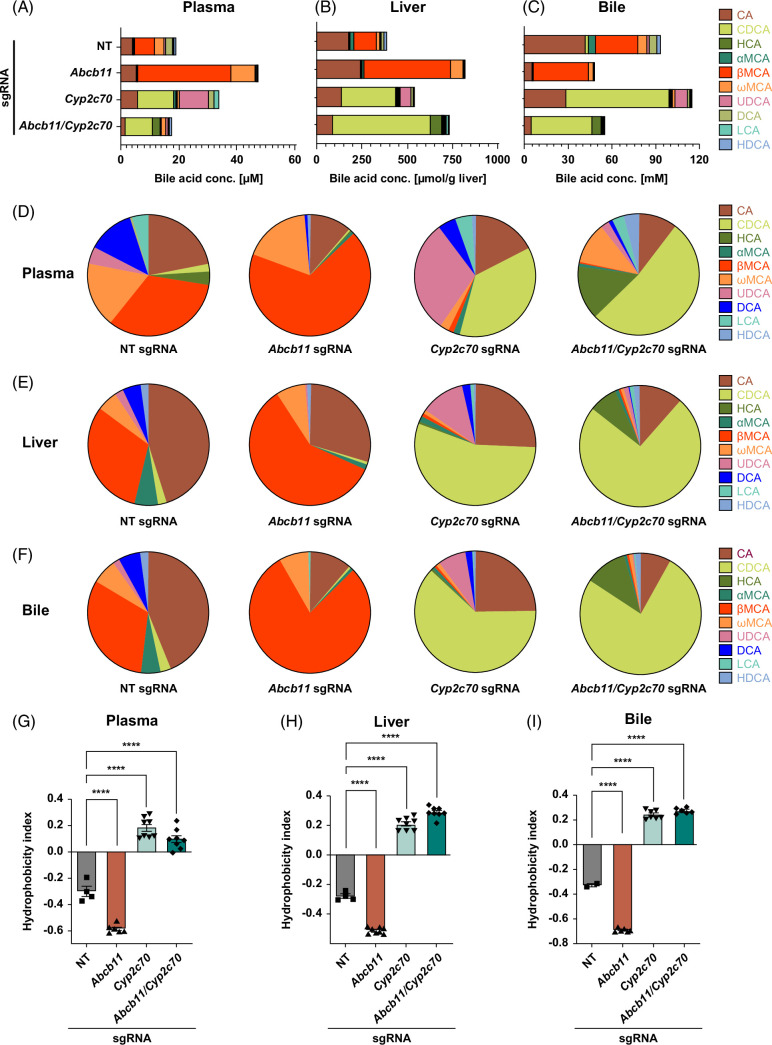
BA profile in male L-Cas9^Tg/Tg^ mice injected with AAV8-*Abcb11* sgRNA, AAV8-*Cyp2c70* sgRNA, or both. Blood, livers, and gallbladder bile collected in Figure 2 were analyzed to evaluate the BA profile. Each species of BAs shown is the sum of unconjugated and conjugated forms. (A–F) The concentration of each BA species (A–C) and the ratio of each BA species to total BAs (D–F) in plasma (A, D), liver (B, E), and gallbladder bile (C, F). (G–I) Hydrophobicity index of BAs in plasma (G), liver (H), and gallbladder bile (I). Data are presented as mean±SEM. ****p < 0.0001 by one-way ANOVA with a post hoc Tukey’s test for multiple comparisons. A representative result from three independent experiments is shown, each exhibiting a similar pattern of results. Abbreviations: AAV8, adeno-associated virus serotype 8; Cyp2c70, cytochrome P450, family 2, subfamily C, polypeptide 70; sgRNA, L-Cas^9Tg/Tg^, liver-specific Cas9^Tg/Tg^; small-guide RNA.

To evaluate the change in BA profile in mice with *Abcb11* and *Cyp2c70* knockdown, BA species in plasma, liver, and gallbladder bile were quantified by the liquid chromatography with tandem mass spectrometry system. More than 99% of BAs were unconjugated, and taurine-conjugated forms of cholic acid, chenodeoxycholic acid, hyocholic acid, αMCA, βMCA, ωMCA, ursodeoxycholic acid, deoxycholic acid, lithocholic acid, and hyodeoxycholic acid. The concentrations of each BA species and the ratio to total BAs in plasma, liver, and gallbladder bile are shown in Figure 3A–F. The absolute values of total BA concentrations differed slightly between the liquid chromatography with tandem mass spectrometry analysis (Figure 3A–C) and the biochemical assay (Figure 2F–H). Still, the trend of each data set was consistent between these 2 assays. Consistent with previous reports on *Cyp2c70-*knockout mice,^11,27^ MCAs were replaced entirely with chenodeoxycholic acid in AAV8-*Cyp2c70* sgRNA administration. Ursodeoxycholic acid and hyocholic acid were uniquely detected in the L-Cas9^Tg/Tg^ mice injected with AAV8-*Abcb11* sgRNA or both AAV8-*Cyp2c70* sgRNA and AAV8-*Abcb11* sgRNA, respectively. The hydrophobicity indices of BAs in plasma, liver, and gallbladder bile indicated that *Cyp2c70* function is indispensable for hydrophilic BA property in mice (Figure 3G–I). Injection of AAV8-*Cyp2c70* sgRNA alone into L-Cas9^Tg/Tg^ mice resulted in ALT elevation (Figure 2D) similar to *Cyp2c70* KO mice,^13,14^ which may be due to the hydrophobic-hydrophilic balance change in the bile acid profile by *Cyp2c70* knockdown.

The phenotypes shown in Figures 2 and 3 were also observed with 6-week-old female L-Cas9^Tg/Tg^ mice (Supplemental Figures 3, 4, http://links.lww.com/HC9/A794).

## DISCUSSION

The present study achieved selective knockdown of the *Abcc2*, *Abcb11*, and *Cyp2c70* genes in mouse liver by *in vivo* genome editing using L-Cas9^Tg/Tg^ mice and sgRNA delivery via AAV8. *Abcc2* knockdown in this system induced jaundice (Supplemental Figure 1, http://links.lww.com/HC9/A794) and phenocopied patients with DJS due to *ABCC2* deficiency.^28,29^ Our method was further able to silence multiple genes simultaneously. In human neonates, *ABCB11* deficiency can cause PFIC2, which leads to cholestasis and progressive cirrhosis in the first decade of life.^9,10^ In contrast to the human disease conditions, however, previous studies have shown that germline knockout of *Abcb11* in mice does not lead to apparent features of liver injury because of the different hydrophobic nature of BA profiles in humans and rodents.^26,30,31^ We simultaneously knocked down the *Cyp2c70* gene with *Abcb11* to mimic the human BA profile. This double knockdown caused cholestasis and hepatocellular damage (Figure 2).

Species-dependent differences in BA composition, especially between humans and rodents, pose a significant challenge for translational research on hepatobiliary diseases. Rodent-specific genes, *Cyp2a12* and *Cyp2c70*, catalyze BA hydroxylation, resulting in a high divergence in the physical properties and physiological activity of BAs between the 2 species. Recently, knockout mice for both genes have been developed and exhibit human-like hydrophobic and cytotoxic BA profiles, providing new research possibilities.^13,14^ In the current study, we successfully humanized the BA profile of L-Cas9^Tg/Tg^ mice using AAV8-*Cyp2c70* sgRNA (Figure 2). Coadministration of AAV8-*Abcb11* sgRNA and AAV8-*Cyp2c70* sgRNA induced more significant hepatic injury in L-Cas9^Tg/Tg^ mice than AAV8-*Abcb11* sgRNA alone (Figure 2E, F). Our platform is a more time-effective and cost-effective alternative to conventional genetically engineered mice (Figure 1), increasing the understanding of BA-related pathogenesis such as Ped-CLD and expanding the potential for translational research.

Asai et al have demonstrated that human-induced pluripotent stem cells (iPSCs) can model cholestatic diseases caused by pathogenic *ABCB11* variants.^32^ Although these iPSC models directly apply to patients with Ped-CLD, the *in vitro* system does not recapitulate physiological BA metabolism. Specifically, the lack of enterohepatic BA circulation makes it difficult to use the iPSC models for preclinical studies of novel therapeutics for Ped-CLD. The current study presents a system in which mice with humanized BA profiles show liver injury due to *Abcb11* deficiency without dietary BA overload. This condition supports the potential application of our system to the preclinical phase of drug development for Ped-CLD.

Recent advances in genetic analysis techniques have led to the discovery of disease-associated genes. By identifying the causative genes, it is possible to distinguish specific patients from a group of patients with similar clinical findings and to analyze the molecular mechanisms involved in the pathogenesis of these patients. Many causative genes for cholestasis and jaundice in children have been identified, such as *JAG1* and *NOTCH2* in Alagille syndrome,^33–35^ *ATP8B1*, *ABCB11*, *ABCB4*, *TJP2*, *NR1H4*, and *MYO5B* in PFIC,^36,37^ *ABCC2* in Dubin–Johnson syndrome (DJS),^28,29^ and *SLC25A13* in neonatal intrahepatic cholestasis caused by citrin deficiency.^38^ Despite the targeted panel sequencing of these known genes, a genetic diagnosis of Ped-CLD has been established in only 25% of cases,^37,39,40^ possibly due to unidentified causative genes for Ped-CLD. Whole exome sequencing and whole genome sequencing are frequently employed to identify novel genes responsible for Mendelian diseases.^41^ These methods generally narrow down candidate genes by identifying pathogenic variants across multiple families with similar phenotypes. However, such an approach is not feasible for ultrarare diseases like Ped-CLD. This study advances *in vivo* genome editing using the CRISPR/Cas9 system to allow the evaluation of hepatic gene function under humanized BA profiles. It provides a platform for experimental validation of the novel candidate genes for Ped-CLD identified from genomic data analysis.

Even in Ped-CLD with known causative genes, there is no common mutation carried by most affected children, and evaluation for novel mutations is usually necessary.^9,42,43^ When genetic testing identifies new rare missense variants in known causative genes, it is challenging to determine pathological and normal variants from genomic data alone. In addition, more than clinical information is required to make this determination because Ped-CLD includes multiple diseases with similar clinical manifestations. Our platform can contribute to this assessment by introducing the genetic variants detected in each patient into mouse orthologs by genome editing. The process from sgRNA design to functional assay can be completed in less than 1 month. Therefore, it can be applied to diagnosing patients with Ped-CLD with new rare variants in known causative genes.

The present study treated L-Cas9^Tg/Tg^ mice with AAV8 sgRNA at 6 weeks. This study design is suitable for assessing gene function in the mature liver, such as hepatobiliary transport involving *Abcc2* and *Abcb11*, but not in the maturation of hepatocytes and bile ducts during the fetal and postnatal periods. The *TJP2* gene, the causative gene of PFIC type 4, encodes tight junction protein 2 (TJP2), a cytoplasmic component of an intercellular junctional complex involved in the maintenance of the structure of bile canaliculi.^44–46^ Disease studies using human iPSC-derived hepatocytes and inducible knockout mice support the contribution of *Tjp2* to hepatobiliary development during the fetal and neonatal periods.^45,47^ In addition to *TJP2*, several other Ped-CLD-causing genes have been identified that may play a role during the fetal and neonatal period, including *MYO5B, LSR, ZFYVE19*, and *KIF12*.^2,36,48^ By delivering AAV8 sgRNA to the fetus, the system established in this study can be applied to evaluating these genes.

In conclusion, we have developed a platform to experimentally validate Ped-CLD candidate genes under conditions that adjusted for species differences in BA metabolism, a significant difficulty in translational research on Ped-CLD. It holds promise for increasing the genetic diagnosis rate of Ped-CLD, elucidating disease pathogenesis, and facilitating the development of novel treatments.

## Supplementary Material

## Data Availability

The data supporting this study’s findings are available from the corresponding author upon reasonable request. Conceptualization, project administration, funding acquisition, and supervision: Hisamitsu Hayashi. Investigation: Kihiro Wakasa, Ryutaro Tamura, Shuhei Osaka, Hajime Takei, Akihiro Asai, and Hiroshi Nittono. Writing—original draft: Kihiro Wakasa, Akihiro Asai, and Hisamitsu Hayashi. Writing—review and editing: KIhiro Wakasa, Akihiro Asai, Hiroyuki Kusuhara, and Hisamitsu Hayashi.
