# Supplementary material for: Rapid in vivo evaluation system for cholestasis-related genes in mice with humanized bile acid profiles
Source: Hepatol Commun. 2024 Mar 22;8(4):e0382. doi: 10.1097/HC9.0000000000000382 (PMC10962888; doi:10.1097/HC9.0000000000000382)
Supplement: Supplementary file 1 [file hc9-8-e0382-s001.docx]

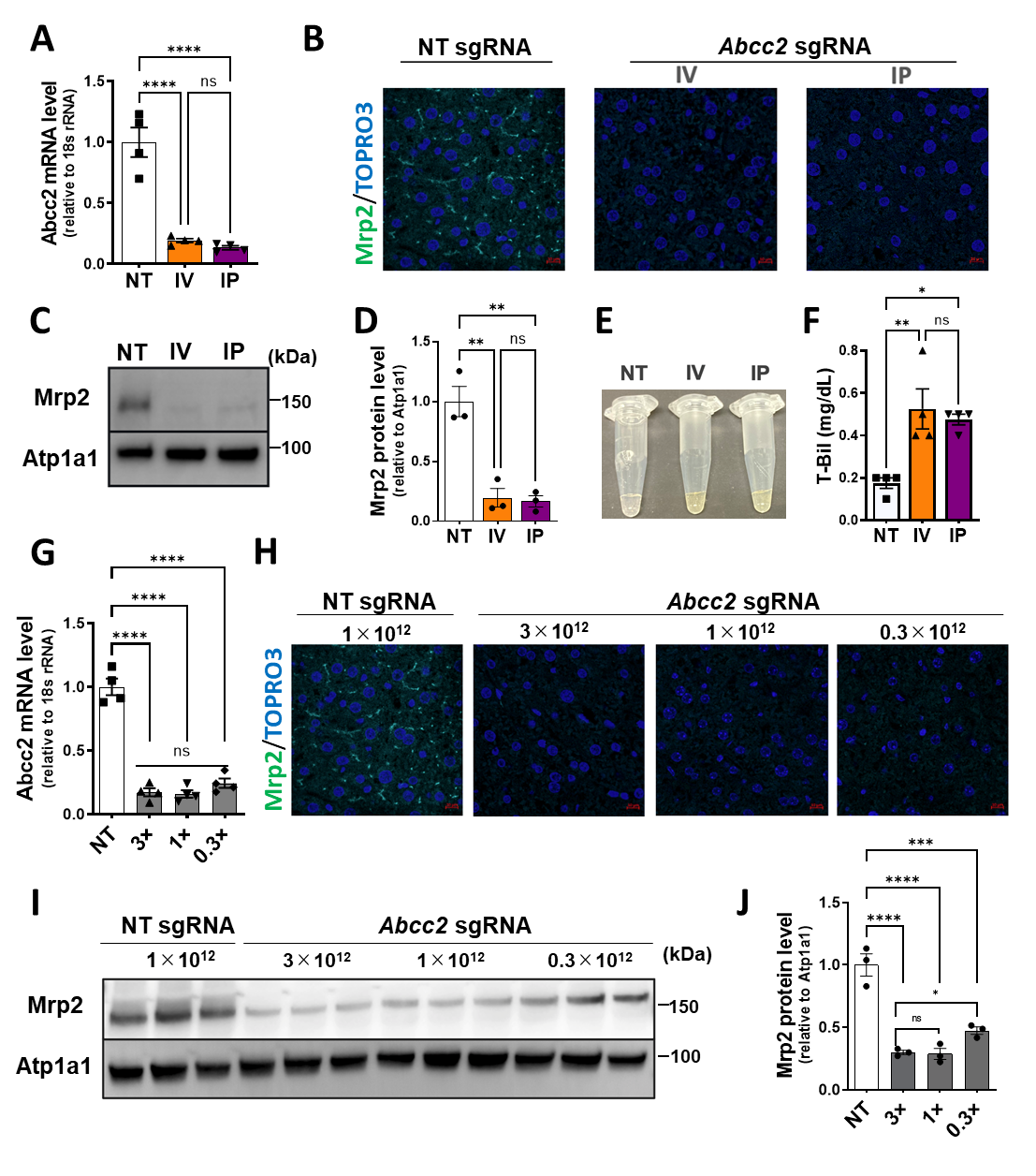


**Supplemental Fig.1. Administration route and dose of AAV8 sgRNA for liver-directed genome editing in L-Cas9^Tg/Tg^ mice.**

**(A–F)** AAV8-NT sgRNA or AAV8-*Abcc2* sgRNA at 1 × 10^12^ genome copies per mouse was administered into male L-Cas9^Tg/Tg^ mice aged 6−7 weeks by intravenous or intraperitoneal injection (n = 4 in each group). Two weeks after the injection, blood and livers were collected and analyzed. **(A)** *Abcc2* mRNA levels in the liver. The mRNA levels are expressed relative to those of 18S rRNA. **(B)** Localization and expression of Mrp2 protein in liver section. Scale bar, 10 μm. **(C, D)** Expression of Mrp2 protein in the crude membrane fraction of the liver. The Mrp2 protein levels are expressed relative to Atp1a1 **(D)**. **(E)** The appearance of plasma. **(F)** Plasma T-Bil levels. **(G–J)** Male L-Cas9^Tg/Tg^ mice aged 6−7 weeks were intraperitoneally injected with AAV8-NT sgRNA or AAV8-*Abcc2* sgRNA at 0.3 × 10^12^, 1 × 10^12^, and 3 × 10^12^ genome copies per mouse. Two weeks after the injection, livers were collected and analyzed. **(G)** *Abcc2* mRNA levels in the liver. The mRNA levels are expressed relative to those of 18S rRNA. **(H)** Localization and expression of Mrp2 protein in liver section. Scale bar, 10 μm. **(I, J)** Expression of Mrp2 protein in the crude membrane fraction of the liver. The Mrp2 protein levels are expressed relative to Atp1a1 **(J)**. In **(A, D, F, G, and J)**, all data are presented as mean ± SEM. **P* < 0.05, ***P* < 0.01, *****P* < 0.0001 by one-way ANOVA with a post hoc Tukey’s test for multiple comparisons. A representative result from three independent experiments is shown, each exhibiting a similar pattern of results. IV, intravenous injection; IP, intraperitoneal injection; NT, non-targeted.


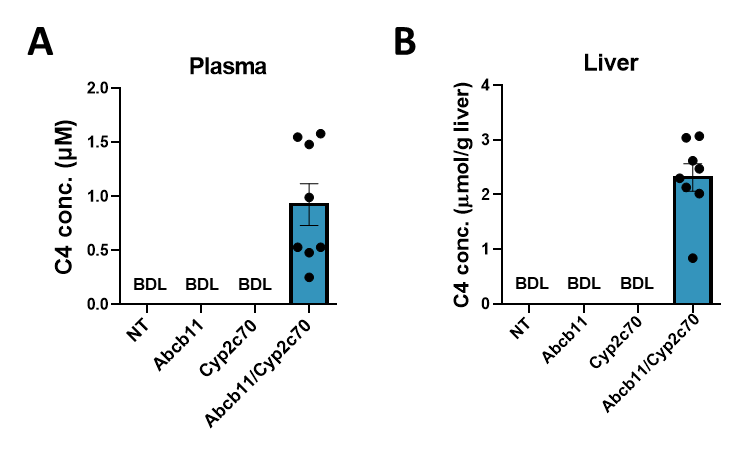


**Supplemental Fig.2. Plasma levels of C4 in male L-Cas9^Tg/Tg^ mice injected with AAV8-*Abcb11* sgRNA, AAV8-*Cyp2c70* sgRNA, or both.**

Blood and livers collected in Fig. 2 were analyzed to evaluate the BA profile. C4(CDCA-Δ4-3-one) levels in plasma (A) and liver (B) are presented as mean ± SEM. A representative result from three independent experiments is shown, each exhibiting a similar pattern of results. BDL, below the detection limit.


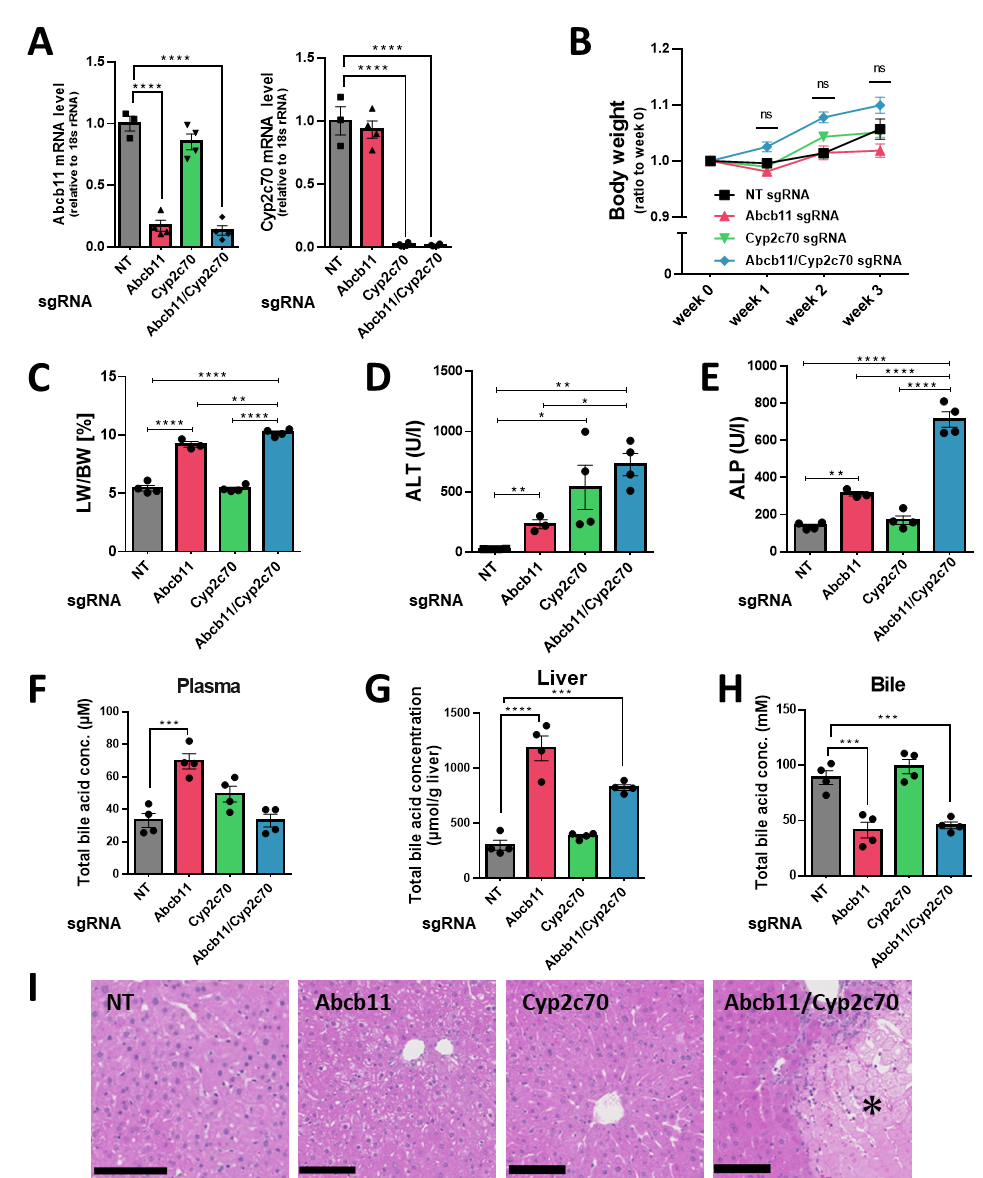


**Supplemental Fig.3. Biochemical and histological analysis of liver function in female L-Cas9^Tg/Tg^ mice injected with AAV8-*Abcb11* sgRNA, AAV8-*Cyp2c70* sgRNA, or both.**

Female L-Cas9^Tg/Tg^ mice aged 6 weeks were intraperitoneally injected with AAV8-NT sgRNA, AAV8-*Abcb11* sgRNA, AAV8-*Cyp2c70* sgRNA, or both AAV8-*Abcb11* sgRNA and AAV8-*Cyp2c70* sgRNA at 1 × 10^12^ genome copies per mouse (n = 4 in each group). Three weeks after the injection, these mice were sacrificed to collect blood, livers, and gallbladder bile. **(A)** *Abcb11* and *Cyp2c70* mRNA levels in the liver. The mRNA levels are expressed relative to those of 18S rRNA. **(B)** Body weight during the experiment. **(C–E)** Liver weight **(C)** and plasma levels of ALT **(D)** and ALP **(E)** at the laparotomy. **(F–H)** Total BA levels in plasma **(F)**, liver **(G)**, and gallbladder bile **(H)**. **(I)** H&E staining of liver section. *, lobular necrosis. Scale bar, 100 μm. In **(A–H)**, all data are presented as mean ± SEM. **P* < 0.05, ****P* < 0.001, *****P* < 0.0001 by one-way ANOVA with a post hoc Tukey’s test for multiple comparisons. A representative result from three independent experiments is shown, each exhibiting a similar pattern of results. BW, body weight; LW, liver weight; NT, non-targeted.


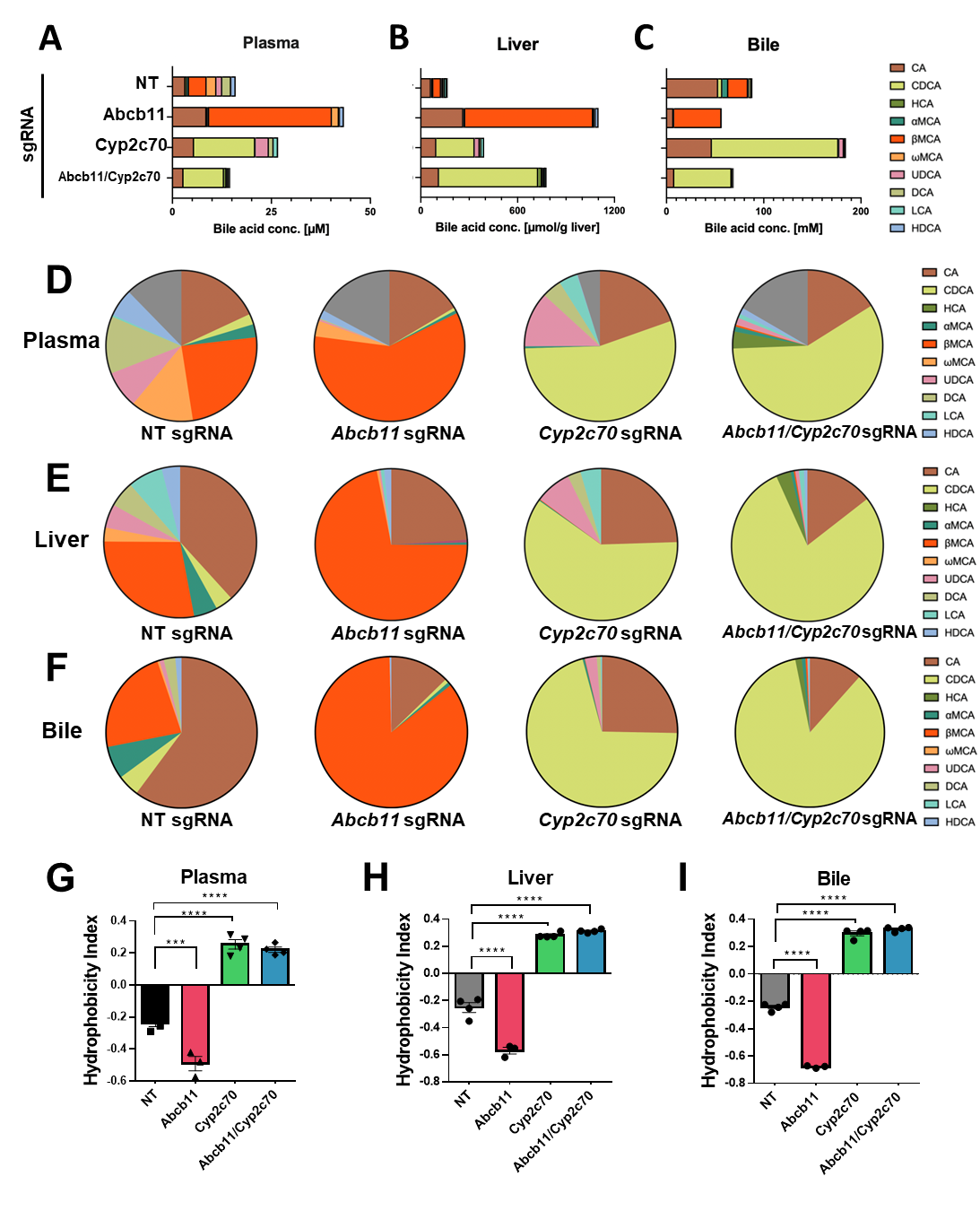
**Supplemental Fig.4. BA profile in female L-Cas9^Tg/Tg^ mice injected with AAV8-*Abcb11* sgRNA, AAV8-*Cyp2c70* sgRNA, or both.**

Blood, livers, and gallbladder bile collected in Supplemental Fig. 3 were analyzed to evaluate the BA profile. Each species of BAs shown is the sum of unconjugated and conjugated forms. **(A–F)** The concentration of each BA species **(A–C)** and the ratio of each BA species to total BAs **(D–F)** in plasma **(A, D)**, liver **(B, E)**, and gallbladder bile **(C, F). (G–I)** Hydrophobicity index of BAs in plasma **(G)**, liver **(H)**, and gallbladder bile **(I).** Data are presented as mean ± SEM. *****P* < 0.0001 by one-way ANOVA with a post hoc Tukey’s test for multiple comparisons. A representative result from three independent experiments is shown, each exhibiting a similar pattern of results.
