# Supplementary material for: Rapid in vivo evaluation system for cholestasis-related genes in mice with humanized bile acid profiles
Source: Hepatol Commun. 2024 Mar 22;8(4):e0382. doi: 10.1097/HC9.0000000000000382 (PMC10962888; doi:10.1097/HC9.0000000000000382)
Supplement: Supplementary file 2 [file hc9-8-e0382-s002.docx]

## Supplementary Table 1. List of reagents

## Supplementary Table 2. List of sgRNA sequences

## Supplementary Table 3. List of antibodies

## Supplementary Table 4. List of primers for qPCR
